# Supplementary material for: Enteric glia regulate Paneth cell secretion and intestinal microbial ecology
Source: bioRxiv. 2024 Apr 19:2024.04.15.589545. Preprint. [Version 1] doi: 10.1101/2024.04.15.589545 (PMC11042301; doi:10.1101/2024.04.15.589545)
Supplement: Supplement 1 [file NIHPP2024.04.15.589545v1-supplement-1.pdf]

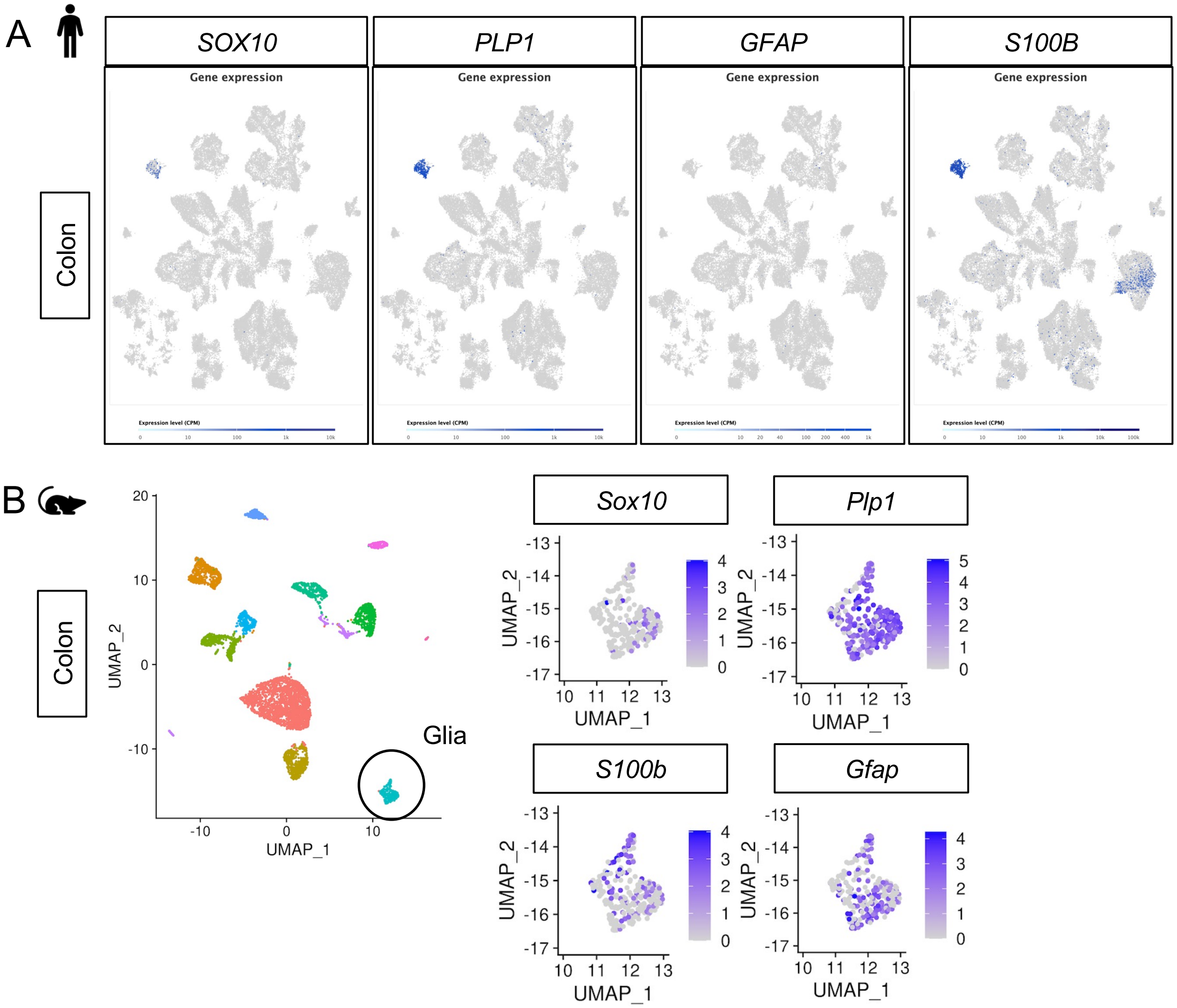

Supplementary Figure 1. Plp1 is the most specific and widely expressed marker of enteric glia in human and mouse colonic mucosa.

A) UMAP plots of 91,103 cells isolated from non-malignant colon tissues from 29 adult colorectal cancer patients [23]. *PLP1* marks most of the cells in the putative glial cluster while no *GFAP* is detected. *S100B* is robustly expressed by glia but also detectable in non-glial cells. *SOX10* is highly specific to the putative glial cluster but is not as universally detected within this cluster as *PLP1* and *S100B*.
B) UMAP plots of Drop-seq data from 3,179 mouse colonic mesenchymal/lamina propria cells [24]. *Plp1* is widely expressed in the putative glial cluster (301 cells) while *Sox10*, *S100b*, and *Gfap* exhibit narrower expression.

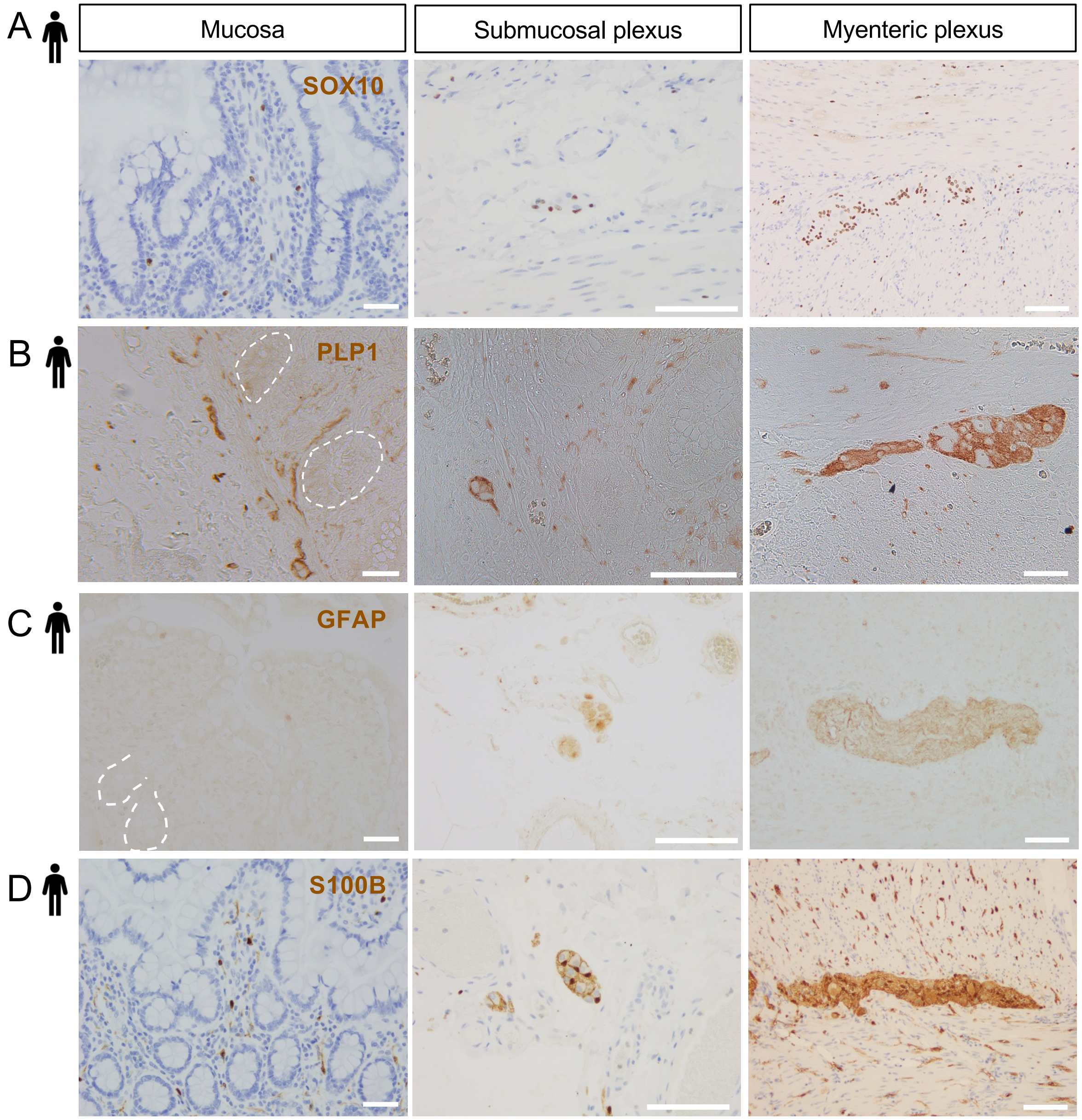

Supplementary Figure 2. S100B, SOX10, PLP1, and GFAP expression across the radial axis of the human small intestine.

A) – D) Representative IHC images of SOX10 (A), PLP1 (B), GFAP (C), and S100B (D) staining in non-diseased small intestinal tissue from adult females shown in three compartments: mucosa (left panels; selected crypts outlined in white for clarity), submucosal ganglia and the surrounding connective tissue and muscle (middle panels), and the myenteric plexus with surrounding circular and longitudinal muscle (right panels). SOX10, PLP1, and S100B were detected in all three compartments. GFAP immunoreactivity was detected in submucosal ganglia (middle panel) and myenteric ganglia (right panel), but not the mucosa. Images are representative of observations made in 5 of 5 subjects for SOX10 and S100B, 3 of 4 subjects for PLP1, and 3 of 3 subjects for GFAP. Scale bars = 100μm (left and right panels) and 50μm (middle panels).

A

Duodenum

Gene	pvalue	padj	log2FoldChange
Aldh3a1	0.000008910	0.1310646	-6.498995
Ighv1-58	0.000014900	0.1460164	-4.029232
Ly6g6c	0.000074400	0.5474189	-4.090178
Sprr1a	0.000103405	0.6086842	-2.120538
Igkv4-91	0.000212050	0.6934495	-2.653247
Nts	0.000239858	0.7059507	-1.333319
Ighv2-6-8	0.000313710	0.8393741	-2.630664
Igkv4-86	0.000399406	0.9400488	-1.288737
Saa3	0.000415216	0.9400488	-2.640961
Tcf23	0.000503807	0.9885368	-1.764401

Ileum

Gene	pvalue	padj	log2FoldChange
Hsd17b6	0.000002430	0.07120339	-0.9071903
Gstm3	0.000006480	0.09471883	-0.8888116
Igkv4-57	0.000014200	0.13850256	-1.7808468
Igkv4-62	0.000107928	0.55743722	-6.1350910
Tiparp	0.000114373	0.55743722	-0.5307053
Slc28a2b	0.000178179	0.74435683	-0.9921444
Nckap5	0.000591408	0.99998609	-0.3574527
Igkv12-38	0.000732130	0.99998609	-5.6854547
Ighv1-14	0.001072487	0.99998609	-5.2105739
Acpp	0.001167716	0.99998609	-0.4302563

Colon

Gene	pvalue	padj	log2FoldChange
Tppp	0.000005720	0.04042812	-0.8680251
Rasef	0.000021700	0.09222872	-0.4437229
Cxadr	0.000094800	0.26185200	-0.4071608
Omp	0.000162683	0.32591658	-0.4957502
Fchsd2	0.000174874	0.32591658	-0.3286969
Ssx2ip	0.000182448	0.32591658	-0.4090985
C730036E19Rik	0.000258691	0.40297592	-4.8994649
mt-Rnr2	0.000397022	0.54803483	-0.6238346
Ipmk	0.000485654	0.55662770	-0.4564745
Ralgds	0.000494894	0.55662770	-0.3194761

B

Duodenum

Gene	pvalue	padj	log2FoldChange
Ighv1-77	0.000001880	0.05546782	2.7875073
Jag2	0.000132530	0.62640252	0.8188377
Cited1	0.000148981	0.62640252	5.6165661
Igkv4-90	0.000202152	0.69344955	1.8245964
Gm42755	0.000458358	0.96360006	5.3594560
BC043934	0.001130218	0.99996906	2.1387513
Esrrb	0.001180178	0.99996906	1.3696598
Defa3	0.002827940	0.99996906	1.8367147
Gm38036	0.003046136	0.99996906	5.5153661
Ighv5-4	0.003383343	0.99996906	1.7316161

Ileum

Gene	pvalue	padj	log2FoldChange
Gm43697	0.000083600	0.5574372	0.6516949
Igkv5-39	0.000310037	0.9999861	2.1554750
Nr2f1	0.000365134	0.9999861	1.6719130
Fam189a2	0.000472555	0.9999861	0.4411970
Efna3	0.000636846	0.9999861	0.7792561
Doc2g	0.000790497	0.9999861	1.1183711
Rpgrip1l	0.000997760	0.9999861	0.5449431
Atp13a2	0.001101490	0.9999861	0.2951110
Gsdma2	0.001394452	0.9999861	1.4361176
Vmac	0.001425860	0.9999861	0.4085882

Colon

Gene	pvalue	padj	log2FoldChange
Lyz1	2.26000e-09	0.000068700	1.3220843
Ighv1-47	2.88000e-08	0.000437645	3.5427220
Naaladl1	6.16000e-06	0.040428115	1.5747556
Olfm4	6.66000e-06	0.040428115	2.2417255
Anpep	1.53000e-05	0.077245808	1.0413319
A530020G20Rik	2.43000e-05	0.092228715	1.0465385
Rbmx	2.74000e-05	0.092504400	0.3842134
Gm8437	4.28000e-05	0.129916521	1.0158035
Cirbp	1.18062e-04	0.298775154	0.6130367
4632404H12Rik	1.45657e-04	0.325916581	0.7008058

Supplementary Figure 3. Changes to whole tissue transcriptome resulting from glial ablation.

A - B) Top ten most down- (A, blue) and upregulated (B, red) genes in the duodenum, ileum, and colon of Cre^{+} mice compared to Cre^{-} mice, ordered by p-value. Differential gene expression analysis was performed using DESeq2.

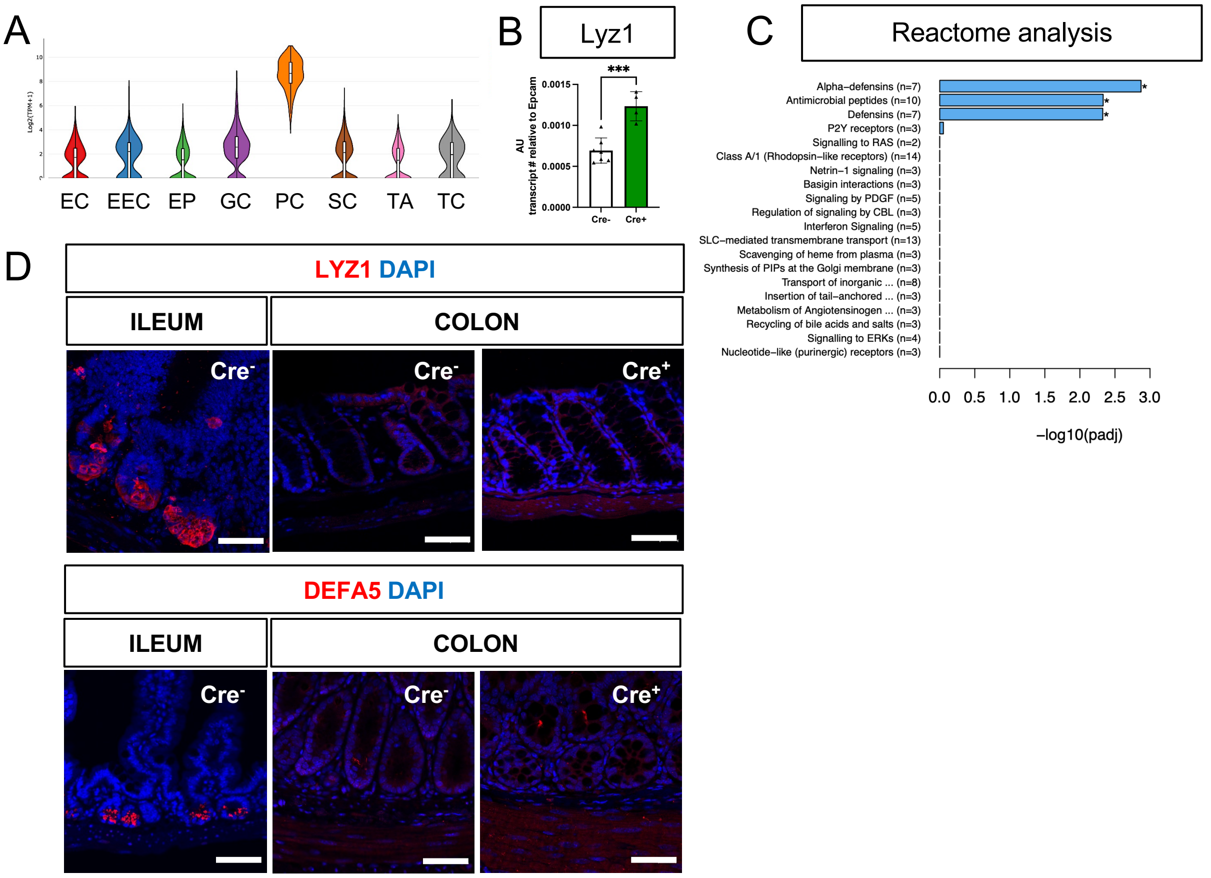

Supplementary Figure 4. Glial ablation induces colonic expression of *Lyz1* at the transcript, but not protein, level.

A) Violin plots of *Lyz1* expression in small intestine epithelial cells show enrichment of expression in Paneth cells (data from scRNAseq of mouse small intestine; [33]). EC – enterocyte, EEC – enteroendocrine cell, EP – enterocyte progenitor, GC – Goblet cell, PC – Paneth cell, SC – Stem cell, TA – Transient Amplifying cell, TC – Tuft cell.
B) Quantitative RT-PCR analysis of *Lyz1* expression in proximal colons of male Cre$^-$ and Cre$^+$ mice. Each data point represents one mouse, with triangles representing males and circles representing females. Error bars represent SEM. ***p<0.001 by unpaired parametric *t*-test.
C) Reactome analysis of differentially expressed genes (significance threshold p<0.05) in colons of Cre$^+$ mice compared to Cre$^-$ mice.
D) Representative IHC images of LYZ1 and DEFA5 staining in the distal ileums of Cre$^-$ mice (positive control) compared to the proximal colons of Cre$^+$ and Cre$^-$ mice (n=3 per genotype). No ectopic LYZ1$^+$ or DEFA5$^+$ cells were detected in the large intestines of glial-depleted mice. Scale bar = 50µm.

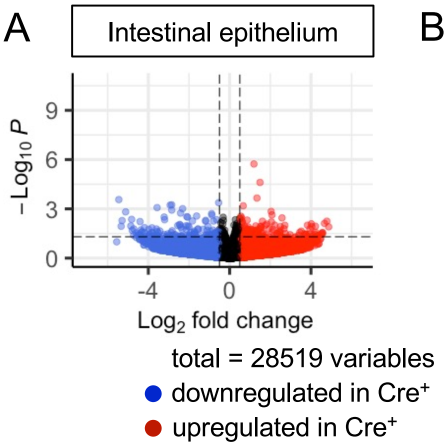

B

Gene	pvalue	padj	log2FoldChange
Gm37144	0.09840504	0.9998656	-0.522150391
Gm19938	0.39241509	0.9998656	-2.937789483
Sox17	0.53205689	0.9998656	-0.782539347
Rb1cc1	0.55996718	0.9998656	-0.057982296
Gm6119	0.68801019	0.9998656	-1.416742266
Gm37277	0.71246509	0.9998656	-0.891106345
Tcea1	0.74707332	0.9998656	-0.031013847
Gm7357	0.78725885	0.9998656	-0.915066669
Xkr4	0.95543068	0.9998656	-0.037170042
Lypla1	0.96654021	0.9998656	-0.004678425

C

Gene	pvalue	padj	log2FoldChange
Fabp1	0.00000186	0.05380245	1.19272539
Slc23a1	0.00002470	0.35717716	1.49261407
Gm37323	0.45266005	0.99986562	2.59490964
Gm6085	0.49856651	0.99986562	1.24846145
Gm6104	0.57713525	0.99986562	1.18660306
Mrpl15	0.69604029	0.99986562	0.04399197
Atp6v1h	0.77116944	0.99986562	0.03639925
Gm2053	0.81285023	0.99986562	0.83534027
Gm38076	0.82284090	0.99986562	0.79119414
Gm6123	0.85799978	0.99986562	0.15977574

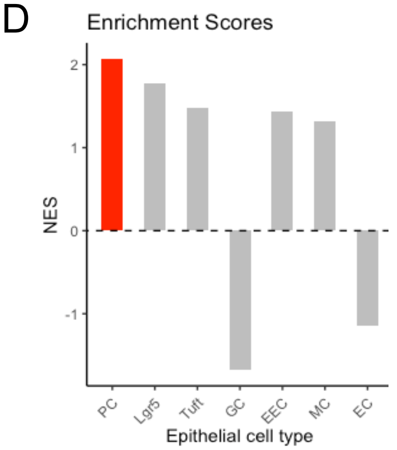

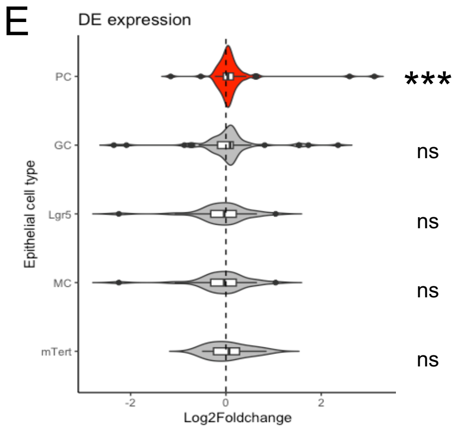

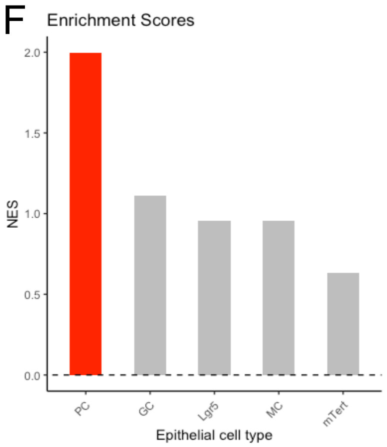

Supplementary Figure 5. Transcriptional profiling of the ileal epithelium in glial-ablated mice reveals enrichment of Paneth cell signatures.

A) Volcano plots showing differentially expressed genes in ileal epithelia of Cre⁻ and Cre⁺ mice. Red and blue colors denote up- and down-regulated genes in Cre⁺ mice compared to Cre⁻ mice with p-value < 0.05, respectively. No genes reached statistical significance cutoff of *padj* < 0.05. Differential analysis was conducted using DESeq2.
B and C) ÅTop ten down- (B) and upregulated (C) genes in the ileal epithelia of Cre⁺ mice compared to Cre⁻ controls, ordered by p-value. Differential analysis was conducted using DESeq2.
D) Related to Main Figure 3B. Normalized enrichment scores from GSEA performed using cell-type specific signatures derived from scRNAseq study of the ileal epithelium ([33], Supplementary Table 1). Red color denotes the significant enrichment consistent across two independent GSEA.
(E) Log2fold change from GSEA of epithelial cell type-specific gene signatures in Cre⁺ vs. Cre⁻ ileal epithelium using cell signatures curated from studies that used transcriptional profiling of individual cell types purified by genetic reporter expression (see Supplementary Table 2 for gene lists). This identifies the Paneth cell signature as most enriched in glial-depleted mice. Thresholds for DE analysis: p-value <0.05. *** p<0.001, FDR<0.001.
(F) Related to (E), normalized enrichment scores from GSEA.

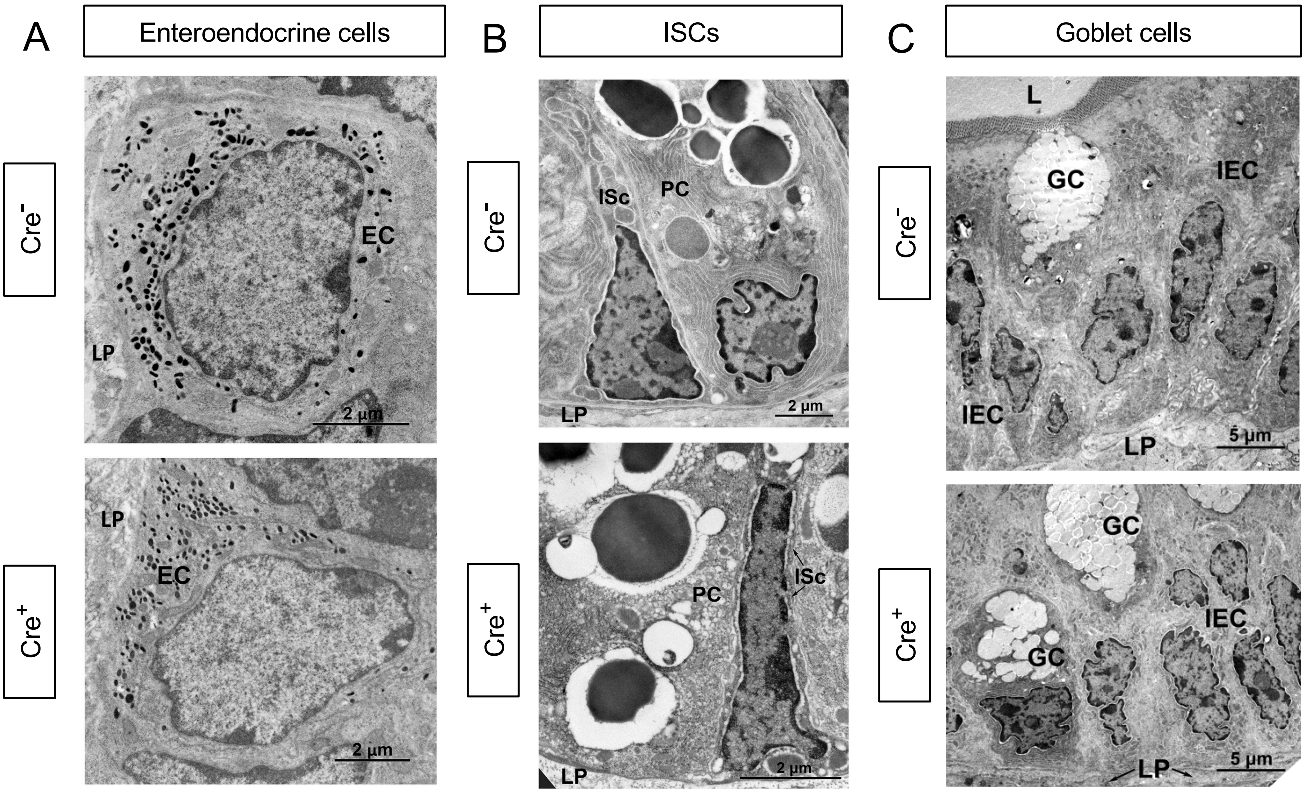

Supplementary Figure 6. Glial depletion does not affect the ultrastructure of enteroendocrine, crypt base stem cells, or goblet cells.

A - C) Transmission electron microscopy of enteroendocrine cells (A; EC), crypt base intestinal stem cells (B; ISc), and goblet cells (C; GC) reveal no changes in the morphology of these cells in 9dpt Cre^{+} mice compared to Cre^{-} littermate controls studied in parallel. Representative images are from n = 2 mice per genotype from independent cohorts. IEC, Intestinal Epithelial Cell; LP, Lamina propria; L, Lumen; PC, Paneth cell. Scale bar = (A-B) 2µm, (C) 5µm.

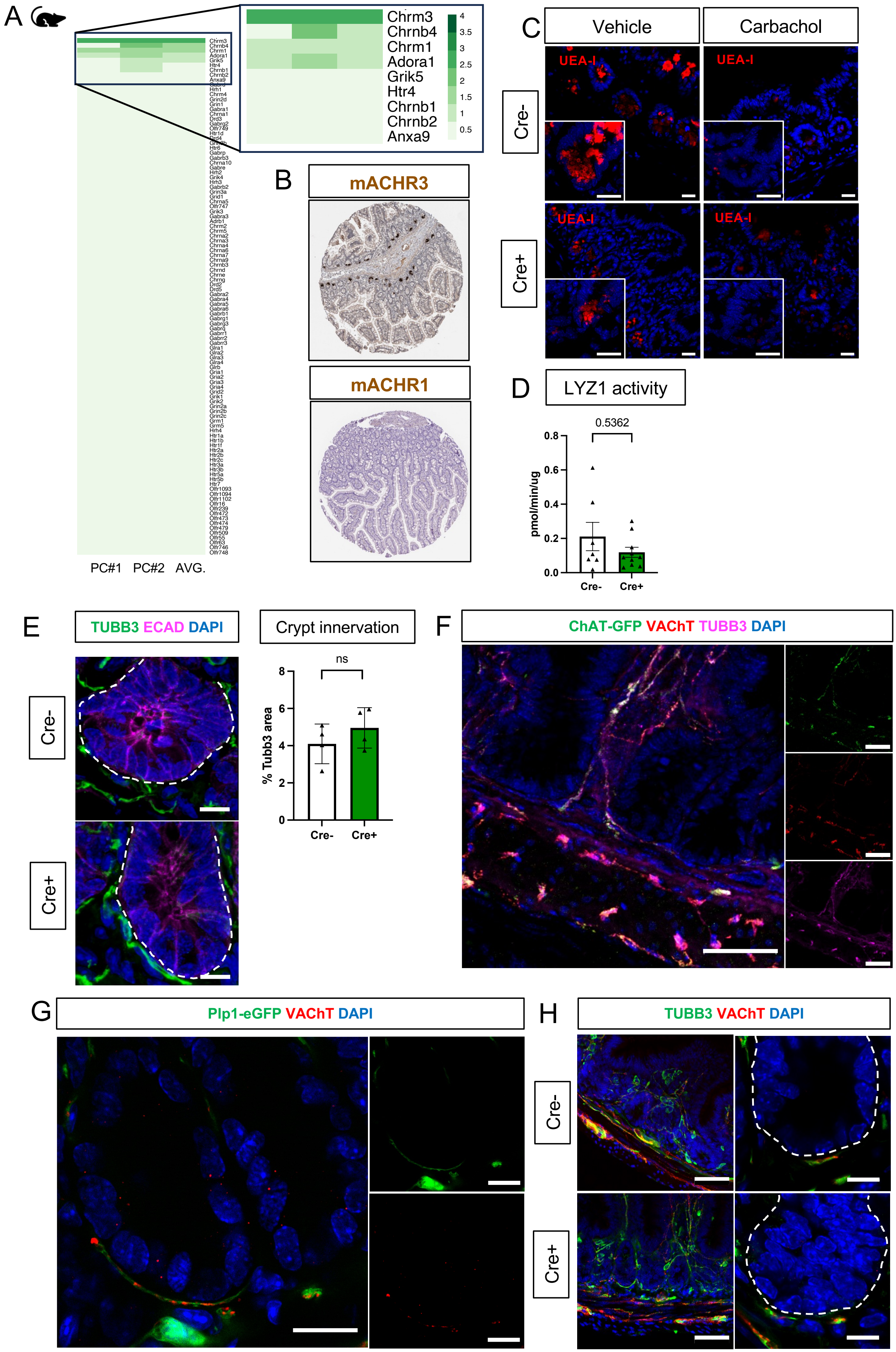

Supplementary Figure 7

Supplementary Figure 7. Depletion of enteric glia does not alter epithelial crypt innervation.

A) Heatmap of neurotransmitter receptor gene expression following GSEA of publicly available RNAseq data from mouse Paneth cells isolated by cell sorting [52] using an Qiagen Ingenuity Pathway Analysis gene set for neurotransmitter receptors. A subset of genes encoding receptors for acetylcholine, specifically Chrm3, Chrnb4, and Chrm1, exhibit the strongest expression in Paneth cells.
B) Representative images of immunohistochemistry for mAChR3 (n=2) and mAChR1 (n=1) in the human small intestine show high expression of mAChR3 (encoded by *CHRM3*) in Paneth cells (Protein Atlas, www.proteinatlas.org).
C) Representative images of cross-sections of ileum from explants isolated from Cre^- and Cre^+ mice, incubated with the cholinergic agonist carbachol (10µM) or vehicle for 30min, and stained with UEA-I (red) to label Paneth cell granules (n=3 mice per condition). Paneth cells in both groups of mice showed robust degranulation of UEA-I^+ granules upon cholinergic stimulation. Scale bar = 20µm.
D) Lysozyme activity in luminal contents of ileal explants from Cre^- and Cre^+ mice following incubation with carbachol (10µM) for 30min. No difference between explants from Cre^- and Cre^+ mice was detected. Each data point represents one mouse (n = 7-10 mice per genotype). Error bars represent SEM. ns – no significant difference in means by Mann-Whitney U test.
E) Representative images of intestinal epithelial crypts from ileal segments of Cre^- and Cre^+ mice immunostained for ECAD (magenta) to label epithelial cell borders and TUBB3 (green) to label nerve fibers. Individual crypt outlined by a dashed line. Quantification of crypt-proximal $TUBB3^+$ neuronal fibers reveals no difference between Cre^- and Cre^+ mice (n = 4 mice per genotype). Error bars represent SEM. ns - no significant difference in means by unpaired parametric *t*-test. Scale bar = 10µm.
F) IHC for VAChT (red) and TUBB3 (magenta) in the ileum of an adult ChAT-GFP mouse. The majority of TUBB3 immunoreactivity around epithelial crypts colocalizes with GFP (green), indicating that cholinergic fibers (green) comprise most of the crypt-innervating fibers. Scale bar = 50µm.
G) IHC for VAChT (red) to label cholinergic fibers in the ileum of an adult PLP1-eGFP mouse in which Plp1-expressing enteric glia are labeled with GFP (green). GFP^+ glia are tightly associated with crypt-innervating cholinergic fibers. Scale bar = 10µm.
H) IHC for TUBB3 (green) and VAChT (red) in the ileums of Cre^- and Cre^+ mice. Individual crypts are magnified in the right panels and outlined by dashed lines. Crypt innervation by fibers labeled with either marker was no different between control and glial-ablated mice. Scale bar = 50µm (left panels) and 10µm (right panels).
Images in F-H are representative of observations made in 3 mice per genotype.

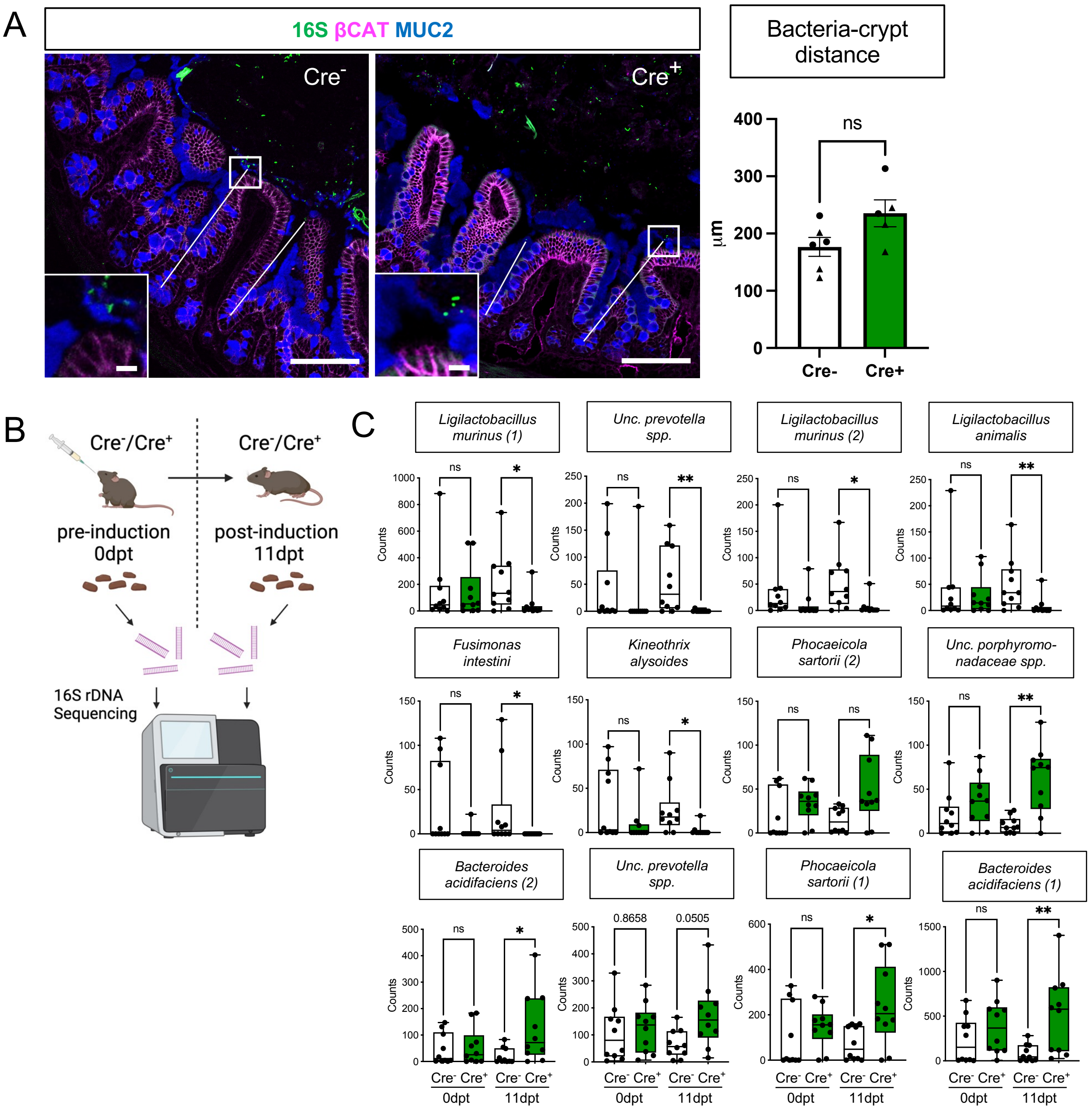

Supplementary Figure 8. Glial depletion alters gut microbial composition but not the spatial relationship between the host and the bacteria. Related to Figure 5.

A) Representative images of 16S rRNA bacterial fluorescent in situ hybridization (green) with IHC for MUC2 (blue) and beta-catenin (magenta) in Cre$^+$ and Cre$^-$ mice to visualize bacteria, mucus, and epithelial cell borders, respectively. The distance of the closest bacterial signal to the center of >50 open crypts/mouse was measured and average values per mouse are shown in the graph. Each data point represents one mouse, with triangles representing males and circles representing females. Error bars represent SEM. ns– non-significant by unpaired t-test. Scale bar = 100μm (large panels) and 10μm (insets).

B) Schematic of experimental design for microbiome analysis. Fecal pellets from Cre$^-$ and Cre$^+$ mice pre- (0dpt) and post- (11dpt) induction were analyzed with 16S rDNA sequencing.

C) Four-way analysis of species identified as differentially abundant between Cre$^-$ and Cre$^+$ mice at 11dpt but not 0dpt (related to Main Fig. 5G). Each data point represents one mouse; both males and females were used. *p<0.05; **p<0.01; ns – non-significant by Dunn's multiple comparison test, following Kruskal-Wallis test.
